# Intraperitoneal pressure measurements in children on peritoneal dialysis: a review and European practice survey

**DOI:** 10.1007/s00467-026-07233-6

**Published:** 2026-03-26

**Authors:** Ariane Zaloszyc, Bruno Ranchin, Alberto Edefonti, Johan VandeWalle, Rukshana Shroff, Claus Peter Schmitt

**Affiliations:** 1 Service de Pédiatrie 1, Hôpital de Hautepierre, Hôpitaux Universitaires de Strasbourg, Strasbourg, France; 2 Pediatric Nephrology Unit, Hôpital Femme Mère Enfant, Hospices Civils de Lyon, Université de Lyon, Lyon, France; 3 Pediatric Nephrology, Dialysis and Transplant Unit, Fondazione IRCCS Ca’ Granda Ospedale Maggiore Policlinico, Milan, Italy; 4 Department of Pediatric Nephrology, Utoped, Universitair Ziekenhuis Gent, Ghent, Belgium; 5 Renal Unit, University College London Great Ormond Street Hospital and Institute of Child Health, London, UK; 6 Medical Faculty Heidelberg, Center for Pediatric and Adolescent Medicine, Heidelberg University, Clinic 1, Heidelberg, Germany

**Keywords:** Peritoneal dialysis, Intraperitoneal pressure measurement, Children, Clinical practice, Intraperitoneal dialysate volume

## Abstract

Prescribing an adequate intraperitoneal dialysate volume is essential in peritoneal dialysis (PD) to balance optimal solute and fluid removal against the risk of increased intraperitoneal pressure (IPP) and to prevent increased reabsorption and mechanical complications. Since the description of manual hydrostatic IPP measurement (IPPM) in children in 1996, a few small observational studies in children and adults have provided limited evidence on the acceptable upper limit of IPP and associated complications. We conducted a practice survey across European pediatric dialysis centers, which showed that of the responding 47 centers, 68% routinely perform IPPM, but the frequency, mode of measurement, accepted upper limit of IPP, and interpretation of IPPM results vary considerably. In this narrative review, we present the current knowledge on IPP and the rationale for a standardized three-point IPPM, a prerequisite to obtain valid scientific data on the relation of IPP with patient outcome, e.g., by means of registries and adequately powered prospective studies. To date, the role of IPPM in improving outcomes for children on PD is uncertain. Advanced, continuous IPPM technologies providing precise, large-scale IPP data together with PD efficiency and tolerability data will improve our understanding of the impact of IPP on patient outcomes.

**Graphical Abstract:**

**Figure:**
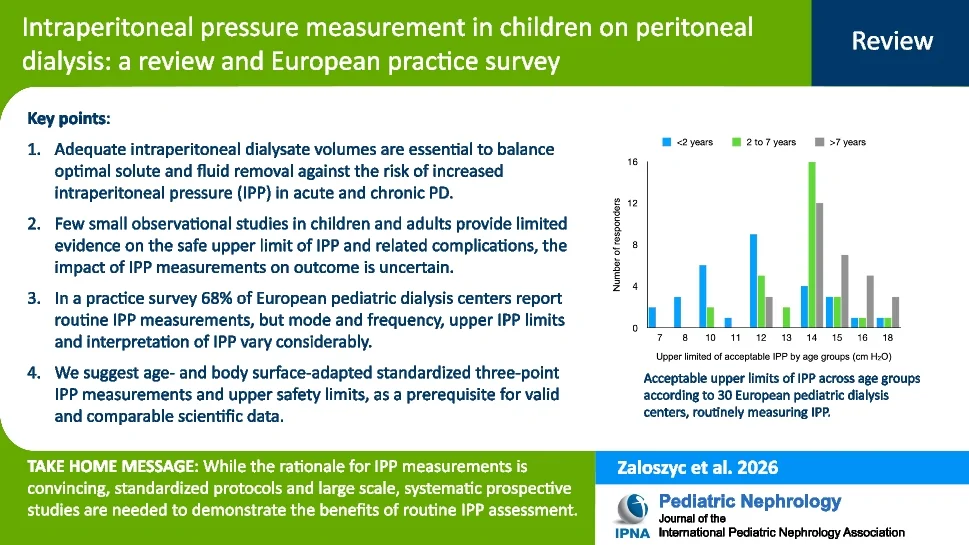
A higher resolution version of the Graphical abstract is available as Supplementary information.

**Supplementary Information:**

The online version contains supplementary material available at https://doi.org/10.1007/s00467-026-07233-6.

## Introduction

Prescribing the optimal dwell volume in growing children on peritoneal dialysis (PD) is a critical challenge for efficient and adequate PD treatment. Increasing the dialysate volume enhances the peritoneal surface area available for solute exchange, and thus for blood purification and fluid removal. On the other hand, prescribing an excessively high dwell volume increases intraperitoneal pressure (IPP), the risk of mechanical complications, such as leaks or hernias, gastro-esophageal reflux and lymphatic reabsorption, which again decreases PD solute clearance and ultrafiltration (UF) [1–4]. Moreover, high IPP may result in patient discomfort, and in critically ill patients may compromise pulmonary function [5–7]. Conversely, prescribing a low dwell volume results in inadequately low clearance and ultrafiltration efficiency. The low dwell volume may create functional hyperpermeability, i.e., a rapid small solute removal and glucose uptake relative to dwell volume; in absolute terms, however, purification and ultrafiltration rates are suboptimal [3, 8–11]. Moreover, a small dwell volume has been linked to an impaired longitudinal growth [12]. Double blinded studies in adults demonstrated that an individual dwell volume of 2, 2.5, and 3 L is not perceived correctly [13, 14].

To provide objective data guiding PD prescription, manual hydrostatic intraperitoneal pressure measurement (IPPM) has been introduced in adults by Durand et al. 33 years ago [15, 16] and adapted for children by Fischbach and colleagues [17, 18]. Practical aspects of IPPM have been detailed in previous publications [1, 16]. The evidence of the impact of routine IPPM on PD patient outcome, however, is scant and clinical practice recommendations have not yet been provided. Although the concept is convincing, routine use of IPPM is debated, as a clear association between IPP and clinical outcomes has not been demonstrated. This narrative review describes current evidence on the factors influencing IPP, the presumed impact on clinical outcomes in pediatric PD patients and the inherent limitations of the current mode of IPPM and how to improve it. A European survey provides an overview on implementation of IPPM in clinical practice.

## Dwell volume and IPP

In children, the optimal dwell volume has been determined empirically and varies according to the child’s age and size from 600 to 1400 mL/m^2^ body surface area (BSA). Lower volumes are prescribed for neonates and infants, due to the higher risk of hernias and leaks. An upper limit of about 800 ml/m^2^ has been suggested for children until the age of 2 years [3, 4, 8, 10]. In adolescents, the dwell volume may be increased stepwise to an upper limit of 1200–1400 ml/m^2^ for the nocturnal exchange in the supine position [3, 8–10, 19], which roughly corresponds to 2–3 L dwell volumes usually applied in adults.

Various small pediatric studies reported mean IPP values relative to dwell volume per m^2^ BSA (Table 1) [1, 20–23]. The IPP ranges from about 3 to 14 cm H_2_O with a mean dwell volume of 600 to 1400 ml/m^2^ (Table 1), with considerable age-dependent, and intra- and interindividual variations [8, 20, 23]. The largely linear increase in IPP in children above 2 years with dwell volumes between 600 and 1400 ml/m^2^ BSA should allow for dwell volume adjustments without exceeding IPP limits, especially if serial, three-point measurements are performed. Of note, there is significant inter-individual variation in IPP for the same BSA standardized dwell volumes, but also significant variability within intra-individual measurements [1, 2, 11, 24]. In adults, a 2 L dwell volume results in an IPP of about 10–16 cm H_2_O [5, 11, 15, 24, 25], with interindividual variations of 5–22.5 cm H_2_O [24].

**Table 1:** IPP according to age and dwell volume per m^2^ BSA

	**Dwell volume** (± 50 ml/m^2^ BSA)	**Intraperitoneal pressure** (cm H_2_O)	**Patients** (n per volume)
**Neonates** (3–6 weeks) 45 measurements Fischbach et al. [20]	600 800 1000 1200 1400	3.0 ± 2.0 4.0 ± 2.0 7.0 ± 1.5 8.0 ± 2.0 11.0 ± 4.0	3 3 3 3 3
**Infants** (< 2 years) 133 measurements Fischbach et al. [20] Sawaya et al. [22]	600 800 1000 1200 1400	5.6 ± 1.9 5.8 ± 1.8 6.4 ± 2.7 6.0 ± 3.0 14.0 ± 4.0	11 10 10 8 8
**Children** (2–18 years) 427 measurements Fischbach et al. [20] Sawaya et al. [22] Zaloszyc et al. [23]	400 600 800 1000 1200 1400	9.5 ± 1.9 9.6 ± 1.7 10.1 ± 2.5 11.2 ± 2.8 12.2 ± 2.0 14.9 ± 2.6	9 25 29 25 18 14

A total of 605 IPPM were performed in 51 children [20, 22, 23]. Mean values of repeated measurements of individual patients were used [22], while from [20] only mean and SD values were available and weighted for respective patient number. Dwell volumes per m^2^ BSA used varied between studies. IPP was measured within 10 min of dialysate infusion in [20, 22] and at the end of the dwell and ultrafiltration considered in [22, 23]. IPP/m^2^ BSA did not differ systematically when measured initially or at the end of the dwell. An upper limit of safe IPP of 14 cm in children above, and 8–10 cm in children below, 2 years of age has been suggested [8, 9, 19, 20, 26].

## Factors influencing IPP beyond IPV

The main factor which influences IPP is IPV, but several other factors impact IPP.

1. Age. Mean IPP/m^2^ BSA dwell volume is lower in neonates and infants than in children until 1000 ml/m^2^ BSA, probably because of the greater elasticity of the thin and more fragile abdominal wall structures (Table 1) [20, 22, 23]. Beyond 1000 ml/m^2^ BSA IPP rapidly increases with higher dwell volumes [1, 9, 20]. In adults, a dwell volume of about 1200 ml/m^2^ BSA results in a higher IPP than in children [27].
2. Gender. In older children and adults, gender may also impact IPP. For a given IPV per m^2^ BSA the mean IPP was reported to be higher in boys than in girls, possibly due to a higher abdominal wall tone in boys [13], although these data are not consistent across studies [24, 28].
3. Body mass index (BMI). Numerous studies in children and adults demonstrate a positive correlation of BMI with IPP, most likely due to the higher intra-abdominal fat. Similarly, BSA, reflecting patient age and BMI, also correlates with IPP [20, 24, 29–31]. In adults, equations based on anthropometric indices predict IPP with good accuracy [32].
4. Organomegaly. Enlarged abdominal organs, such as hepato-splenomegaly in children with portal hypertension and nephromegaly in patients with polycystic kidney disease, increase IPP. In adult PD patients, height normalized kidney mass correlated with IPP [29].
5. Posture. The position of the patient influences IPP. For the same dwell volume, IPP is higher when the patient is in the upright than in the supine position [33, 34], and highest in the sitting position [7]. In six children, two 90-min peritoneal equilibration tests (PET) (1000 ml/m^2^; 1.36% glucose), the first one in supine, the second one in upright position demonstrated an IPP of 8 ± 2.4 cm H_2_O in the supine and 18.4 ± 4.8 cm H_2_O in the upright position, i.e., a 130 ± 35% higher IPP [33]. Creatinine, urea and phosphate removal were faster in the supine position, while UF was not affected; the underlying mechanisms are uncertain.
6. PD vintage. IPP declines with time after PD catheter insertion, presumably due to lower pain perception. It further declines over several months to years with adaptation of the peritoneal cavity to the IP volume [15, 20, 34, 35].
7. PD fluid composition. Higher IPP has been reported with acidic PD fluids, containing high concentrations of glucose degradation products (GDP), than with neutral pH, low GDP fluids [36]. One observational study in 21 children (6 on icodextrin) suggests lower IPP with icodextrin versus glucose-containing PD fluids; the underlying mechanisms are uncertain [22].

Altogether, the various factors modifying IPP illustrate the complex interplay of physiological and PD- and non-PD-related pathophysiological mechanisms in children and provide the rationale that IPP should not be derived from simple volumetric considerations but quantified with an accurate method.

## IPP and clinical outcome

IPP is difficult to perceive, and patient estimates are imprecise [13, 14]. An IPP above 18 cm H_2_O, however, may induce clinical signs of discomfort and even pain in pediatric and adult patients [4, 8, 15, 19]. In an observational study in 61 adult PD patients, an IPP exceeding 14 cm H_2_O was associated with an increased risk of enteric peritonitis [24]. In 52 adults, an IPP above 13 cm H_2_O independently predicted the composite end point of switch to hemodialysis or death [28]. Sixteen adult PD patients with an IPP ≥ 20 cm had more hernias and leaks than 33 patients with a lower IPP [34]. In contrast, in a population-based study in Taiwan, comprising 6928 adult PD patients of which 530 developed hernias, maximal dwell volume did not predict hernia development, but IPP was not determined [37]. This indicates first that the risk for hernia cannot be controlled by accounting for the body size-adjusted dwell volume only.

In school children on CAPD, those who developed hernias (*n* = 7) had a higher IPP of 10.9 ± 2.6 cm compared to those who did not develop hernias (IPP 8.1 ± 2.6 cm H_2_O; *n* = 7; *p* = 0.003) [27]. In 8 children, increasing dwell volume from 800 to 1400 and 2000 ml/m^2^ BSA linearly increased IPP from 8.4 ± 1.4 to 12.1 ± 1.4 cm and 18.3 ± 1.4 cm H_2_O together with a continuous increase in mass area transfer coefficient for urea, and with the highest IPV a plateau for creatinine and a decline for phosphate, together with clinical discomfort [8, 20]. Especially in critically ill children with AKI, a high IPP may compromise pulmonary function [6].

Three studies with 33, 8, and 23 adult PD patients demonstrated a negative correlation of IPP with UF rate [25, 34, 38]. High IPP increases reabsorption of fluid and solutes [8, 11, 19, 20, 39, 40]. To a certain individual limit, the negative impact of high IPP should be counterbalanced by an enhanced peritoneal surface area recruited for clearance by the large IPV and a higher time-integrated blood dialysate solute gradient achieved with the large IPV [11, 19, 33, 38, 39]. In 62 adults, an increased IPP was an independent risk factor for residual kidney function (RKF) loss [41]. An increase in IPP of 1 cm H_2_O increased the risk of rapid RKF decline by 1.68-fold, most likely due to IPP-induced reduction in kidney perfusion [41–43]. Best RKF preservation was observed with an IPP < 15.7 cm H_2_O [41]. Studies in vitro and in mice suggest pressure-induced, CD44-mediated peritoneal fibrosis [44]; in how far such findings are relevant in humans is unknown. In ICU patients, according to the World Society of Abdominal Compartment Syndrome, intra-abdominal hypertension is defined as a sustained elevation of IPP ≥ 12 mmHg, i.e., 16.3 cm H_2_O, and abdominal compartment syndrome is defined as a sustained IPP > 20 mmHg, i.e., 27.2 cm H_2_O, which is associated with organ dysfunction or failure [45, 46].

Altogether, these studies suggest a significant impact of IPP on PD efficacy and tolerability. For an overview please also see Supplemental Table 1. Based on these studies, an upper safe IPP limit of 14 cm H_2_O has been suggested for children above 2 years of age, and of 8–10 cm H_2_O and a maximal dwell of 800 ml/m^2^ in children below 2 years [8, 9, 19, 20, 26]. Whether routine measurement of IPP results in improved patient outcomes, i.e., optimized efficiency, fewer mechanical and infectious complications and better PD sustainability, is currently unknown.

## Limitations and risks of current IPPM practice

IPPM is a simple procedure, but thorough standardization is mandatory, and results depend on good patient cooperation, which may be challenging, especially in young children. Numerous factors influence IPP, and require consideration, such as micturition and defecation prior to IPPM, patient fear and pain. Correct, pressure-free connection of a tube and bag system is mandatory. UF rate, and thus IPP, vary considerably between individuals. If dwell time exceeds 5–10 min, UF may have already occurred, increasing IPP. IPP may be measured at the end of the dwell and related to effluent volume, i.e., dwell volume plus UF, but this does not allow interindividual comparison of IPP relative to a standard dwell volume (e.g., 1 l/m^2^ BSA). These factors may result in inaccurate and falsely high IPP, which may not be captured on IPPM, especially if only a single dwell volume measurement is performed. Three-point measurements with dwell volumes standardized per m^2^ BSA, resulting in a linear increase in IPP, indicate a technically more precise measurement and describe the individual abdominal distensibility. The correlation of IPP with intraperitoneal dialysate volume should be stronger if serial, BSA-standardized IPP measurements with increasing dwell volumes are used, compared to single-point measurements with actual, individual dwell volumes [1, 20]. We therefore suggest using three-point measurements with 600, 1000 and 1400 ml/m^2^ BSA dialysate volumes in children above 2 years of age (and no increased risk of hernias and leak) resulting in a linear relationship with IPP. Average IPP values related to such dwell volumes have been published for comparison (Table 1), but data based on more children and including three-point measurements in infants with lower dwell volumes (e.g., 200, 400, 600 ml/m^2^ in newborns and 300, 600 and 900 ml/m^2^ BSA in infants) are needed. Despite rigorous IPPM execution, inter-individual variability may be high and repeated measurements may be needed in some patients [22].

Risks associated with IPPM are low. It requires an additional patient connection, which may also be used for a subsequent PET. This is associated with a low risk of infection. Since the patient is relaxed in supine position and IPP is measured stepwise with increasing fill volumes, the risk of mechanical complications and of discomfort is very low. The procedure takes about 20 min but requires patient cooperation and trained dialysis nurses.

## Survey on IPPM implementation and practice in European Pediatric dialysis centers

To gain insights into current clinical practice and experience with IPPM we performed a survey in European pediatric dialysis centers.

### Methods

Paediatric nephrologists from centers which are members of the European Rare Kidney Disease Reference Network (ERKNet), the Société de Néphrologie Pédiatrique (SNP), the European Society of Pediatric Nephrology (ESPN) Dialysis Working Group, and the European Pediatric Dialysis Working Group (EPDWG) were invited in October 2024 by e-mail to reply to 21 questions related to IPPM via an online questionnaire (see supplemental data) and a reminder in May 2025. Sixty-two out of 92 pediatric nephrologists contacted, from 19 countries, replied; 28 of 58 centers contacted by ERKNet, 32 out of 50 contacted via the ESPN/EPDWG and 13 out of 28 SNP centers (SNP), with most centers being part of more than one organization. Two replies from physicians in adult units, 2 from two non-European centers (Algeria and Morocco) and 11 repeated center replies were excluded. The study was reviewed and approved by the Strasbourg local IRB (CE-2025–85). Only one answer per center was included; in case more replies were obtained, the center was contacted.

### Results

The 47 center responses were from 16 countries: 14 from France, 8 from Italy, 5 from the United Kingdom, 3 each from Spain and the Netherlands, 2 each from Belgium, Finland and Germany, and one each from Austria, Greece, Lithuania, Luxembourg, Poland, Portugal, Turkey and Slovenia. Personal dialysis experience was 16.6 ± 9.6 years. Eight doctors had ≤ 5 years of experience in dialysis, 9 between 6 and 10 years, 12 between 11 and 20 years and 16 had more than 20 years of experience. Two colleagues did not reply to this question. The median number of PD patients per center was 3, range 0–30.

### IPPM implementation in clinical practice

Fifteen of the 47 centers (31.9%) did not measure IPP. Reasons (multiple answers) included the following: lack of experience (11), lack of evidence (7), lack of both (5), lack of time (4), lack of time and experience (1). Two centers stated that they should/will do it. 32 responding centers stated to perform IPPM (68.1%), 13 from France and 19 from 13 other countries. 26 centers have a local written protocol, two (one from German and one from Spain) refer to national standards of IPPM.

### IPPM procedure

Twelve centers use a specifically manufactured visual scale system and 20 other systems for IPPM (such as self-made scales or central venous pressure measurement kits). Most centers use double chamber PD fluids, with all three different glucose concentrations, 9.4% icodextrin (Suppl. Table 1). Likewise, frequency and timing of IPPM varied substantially across centers. Twenty-four out of the 32 centers (75%) where IPP is determined, perform routine measurements. Nine of these centers measure IPPM every 6 months, one center every 4 months, seven every 3 months, two monthly, one center yearly, and four centers with variations according to individual needs. Of the eight centers not measuring IPPM on a regular basis, 7 (87%) measure IPP in case of poor dwell volume tolerance noted by the patient/caregivers, 6 (75%) in case of PD-related complications, 5 (62%) in case of low PD efficiency, 5 (62%) at start of PD, and 1 (12%) before increasing dwell volume.

Single-point IPPM is performed in 17 (53%) centers, and serial IPP measurements with increasing IP volumes in 15 (47%). Absolute dwell volumes (e.g., actual day/night-time dwell volume) are used in 13 (42%) of the centers, while 16 (52%) of the centers applied increasing dwell volumes per BSA (e.g., 600/1000/1400 ml/m^2^). Both absolute and BSA standardized dwell volumes were used in 2 centers, 1 center did not answer. The dwell is specifically intended for IPPM in 16 centers (52%) and combined with other measurements (mostly with PET) in 10 centers, 5 centers reported to do separate IPPM and combined with another measurement, 1 center did not answer. IPPM is measured within 10 min after infusion in 17 centers, at the end of the dwell in seven, and at both timepoints in eight centers.

**Fig. 1:**
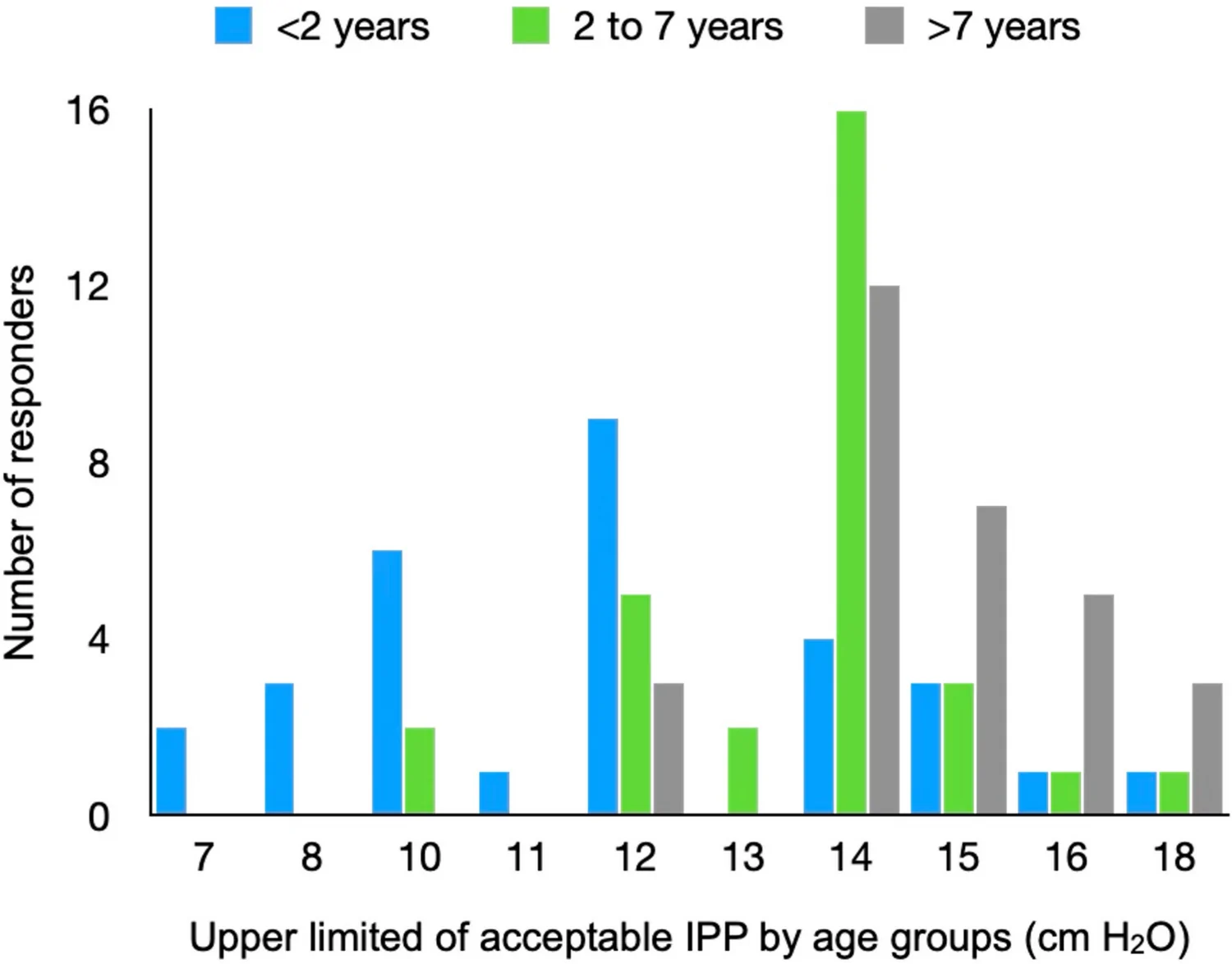
Upper limits of IPP for infants, children 2–7 and above 7 years of age, considered acceptable for PD (according to 30 European pediatric dialysis centers)

Twenty-seven of the 32 centers (84%) performing IPPM in clinical practice adapt the dwell volume prescription to the IPP; 5 centers did not answer the question. Nineteen of the 27 centers (70%) consider age- or body size-related upper limits of acceptable IPP for PD prescription. The age group-dependent upper limit of acceptable, absolute IPP varies substantially between centers (Fig. 1), with a mean upper IPP of 11.8 ± 2.7 (range 7–18) cm H_2_O for infants, 13.7 ± 1.6 (10–18) cm H_2_O for children between 2 and7 years and 14.8 ± 1.6 (12–18) cm H_2_O for children above 7 years of age.

Eighteen out of 19 centers (95%) consider an upper limit of IPP for PD prescription, i.e., adapted the PD regimen accordingly. 15 (79%) reduce dwell volume when IPP is above their upper limit, 8 (42%) increase dwell volume with IPP below their upper limit, and 4 (21%) change the dwell volume only based on two independent IPPM. Twenty-nine out of 32 responders (91%) stated that IPPM has an impact on patient outcome (Table 2).

**Table 2:** Impact of IPPM on patient outcomes

**Impact of IPPM on patient outcome**	*N* = 32 (%)
It improves PD efficiency	23 (72%)
It reduces PD related complications (such as hernias, leakages)	11 (34%)
It improves compliance	5 (16%)
It improves confidence of caregivers/family	5 (16%)
No impact	3 (10%)

*IPPM* intraperitoneal pressure measurement, *PD* peritoneal dialysis

## Discussion and conclusions

Experienced European pediatric nephrologists from 47 pediatric dialysis centers provided important information on current implementation of IPPM. While one-third of respondents do not measure IPP due to lack of experience, time, or evidence, two-thirds have implemented IPPM in clinical practice. IPPM has been introduced in adults by the French colleagues Durand et al. [15, 16] and for children by Fischbach et al. [17, 18]. This may explain the high acceptance rate of IPPM in France. The large number of French respondents may further be explained by the high number of pediatric PD centers in France of 24 and that these centers were also contacted via the national society. Other limitations of the survey are the selection bias inherent to the fact that only centers active in the four organizations were involved, i.e., of active centers, possibly more prone to perform IPPM and lacking responses to some of the questions.

There is a considerable lack of IPPM standardization across centers regarding measurement devices, frequency, and timing of the measurement. The acceptable upper limit of IPP considered in the centers substantially varies in all three age groups, precluding consistent conclusions. In line with this, the survey demonstrates a variety of different conclusions for PD prescription and of the estimated impact on patient outcome. These data show the urgent need for standardization of IPPM over Europe and for prospective—and at best randomized—trials, aimed at demonstrating efficacy and cost effectiveness of IPPM.

A recent editorial comment to a publication on a novel equation for estimating IPP in PD [32], suggests that IPP should be measured on a routine basis in adult PD patients, at least at the beginning of PD, and whenever clinically indicated, in order to detect abnormalities and to prevent potential complications [47]. The 2020 ISPD Practice Recommendations for Prescribing High-Quality Goal-Directed PD does not discuss IPPM [48]. A subsequent commentary on these recommendations mentions IPPM in pediatric PD as a possible tool to guide adjustment in dwell volume, referring to a review by Fischbach and colleagues [10], while pointing out that it is unclear how this is practiced in pediatric dialysis units [49]. While the latter finds answer in our European survey, it remains elusive as to how far IPPM improves PD quality.

Taken together, experimental and clinical studies suggest an impact of IPP on PD solute clearance and UF [40] and on patient comfort [4, 8, 15, 19]. The utility of IPPM in clinical practice is based on limited observational data. Especially in growing children with considerable variations in IPP, IPPM, performed on a regular basis, may inform dwell volume prescription in clinical practice. Our survey, however, demonstrates striking heterogeneity regarding IPPM devices, frequency and timing of IPPM, target IPP and conclusions for PD prescription. Standardization of IPPM practice across centers and countries is a critical unmet need in pediatric PD and should be followed by large-scale prospective studies to address the yet uncertain impact of IPPM on relevant clinical endpoints [31], i.e., PD efficiency, tolerability and sustainability. After discussion of the survey in the EPDWG and at several conferences, the authors of this publication suggest performing standardized three-point measurements with age-adapted fill volumes (see above), as a prerequisite to obtain valid and comparable scientific data. The latter is needed to justify the additional time, costs and workload associated with IPPM. Moreover, an upper limit of IPP of 14 cm H_2_O in children above 2 years of age, and of 8–10 cm in infants are suggested as safety margins, based on limited observational evidence and almost three decades of experience with IPPM in pediatric patients [8, 9, 19, 20, 26]. Based on this standardized approach data should be systematically collected, e.g., in the International Pediatric Peritoneal Dialysis Network (https://pedpd.org/).

Since manual IPP measurements with a simple tube system visually adapted to the mid axillary line are prone to inaccuracy, automated devices (continuously) measuring IPP should be developed. Recently, Zhu et al. published a model of the relationship between IPP and IPV in 13 adults using cycler-integrated pressure sensors for continuous IPP measurement [50]. They provide accurate and precise IPP measurements and important physiological insights into the complex IPP dynamics related to small solute clearance and ultrafiltration.

## Supplementary Information

Below is the link to the electronic supplementary material.

ESM 1 Graphical abstract (PPTX 128 KB)

ESM 2 (DOCX 42.4 KB)

## References

[1] Fischbach M, Terzic J, Laugel V, Escande B, Dangelser C, Helmstetter A (2003) Measurement of hydrostatic intraperitoneal pressure: a useful tool for the improvement of dialysis dose prescription. Pediatr Nephrol 18:976–980. 10.1007/s00467-003-1199-9 12898379

[2] Durand PY (2000) Optimization of fill volumes in automated peritoneal dialysis. Perit Dial Int 20:601–602 11216546

[3] Fischbach M, Stefanidis CJ, Watson AR, European Paediatric Peritoneal Dialysis Working Group (2002) Guidelines by an ad hoc European committee on adequacy of the paediatric peritoneal dialysis prescription. Nephrol Dial Transplant 17:380–385 10.1093/ndt/17.3.380 11865081

[4] Fischbach M, Warady BA (2009) Peritoneal dialysis prescription in children: bedside principles for optimal practice. Pediatr Nephrol 24:1633–1642. 10.1007/s00467-008-0979-7 PMC2719743 18807074

[5] Durand PY, Chanliau J, Gamberoni J, Hestin D, Kessler M (1994) APD: clinical measurement of the maximal acceptable intraperitoneal volume. Adv Perit Dial 10:63–67 7999866

[6] Bunchman TE, Meldrum MK, Meliones JE, Sedman AB, Walters MB, Kershaw DB (1992) Pulmonary function variation in ventilator dependent critically ill infants on peritoneal dialysis. Adv Perit Dial 8:75–78 1361858

[7] Twardowski ZJ, Prowant BF, Nolph KD, Martinez AJ, Lampton LM (1983) High volume, low frequency continuous ambulatory peritoneal dialysis. Kidney Int 23:64–70. 10.1038/ki.1983.12 6834695

[8] Fischbach M, Terzic J, Menouer S, Haraldsson B (2000) Optimal volume prescription for children on peritoneal dialysis. Perit Dial Int 20:603–606 11216547

[9] Fischbach M, Haraldsson B (2001) Dynamic changes of the total pore area available for peritoneal exchange in children. J Am Soc Nephrol 12:1524–1529. 10.1681/ASN.V1271524 11423582

[10] Fischbach M, Zaloszyc A, Schaefer B, Schmitt CP (2014) Optimizing peritoneal dialysis prescription for volume control: the importance of varying dwell time and dwell volume. Pediatr Nephrol 29:1321–1327. 10.1007/s00467-013-2573-x 23903692

[11] Perez Diaz V, Sanz Ballesteros S, Hernandez Garcia E, Descalzo Casado E, Herguedas Callejo I, Ferrer Perales C (2017) Intraperitoneal pressure in peritoneal dialysis. Nefrologia 37:579–586. 10.1016/j.nefro.2017.05.014 28739249

[12] Schaefer F, Klaus G, Mehls O (1999) Peritoneal transport properties and dialysis dose affect growth and nutritional status in children on chronic peritoneal dialysis. Mid-European Pediatric Peritoneal Dialysis Study Group. J Am Soc Nephrol 10:1786–1792. 10.1681/ASN.V1081786 10446947

[13] de Jesus Ventura M, Amato D, Correa-Rotter R, Paniagua R (2000) Relationship between fill volume, intraperitoneal pressure, body size, and subjective discomfort perception in CAPD patients. Mexican Nephrology Collaborative Study Group. Perit Dial Int 20:188–193 10809242

[14] Harris KP, Keogh AM, Alderson L (2001) Peritoneal dialysis fill volume: can the patient tell the difference? Perit Dial Int 21(Suppl 3):S144-147 11887809

[15] Durand PY, Chanliau J, Gamberoni J, Hestin D, Kessler M (1996) Measurement of hydrostatic intraperitoneal pressure: a necessary routine test in peritoneal dialysis. Perit Dial Int 16(Suppl 1):S84-87 8728169

[16] Durand PY, Chanliau J, Gamberoni J, Hestin D, Kessler M (1992) Routine measurement of hydrostatic intraperitoneal pressure. Adv Perit Dial 8:108–112 1361762

[17] Fischbach M, Desprez P, Donnars F, Geisert J (1994) Hydrostatic intraperitoneal pressure in children on peritoneal dialysis: practical implications. An 18-month clinical experience. Adv Perit Dial 10:294–296 7999848

[18] Fischbach M, Desprez P, Terzic J, Lahlou A, Mengus L, Geisert J (1996) Use of intraperitoneal pressure, ultrafiltration and purification dwell times for individual peritoneal dialysis prescription in children. Clin Nephrol 46:14–16 8832143

[19] Fischbach M, Terzic J, Gaugler C, Bergere V, Munch K, Hamel G, Provot E, Donnars F (1998) Impact of increased intraperitoneal fill volume on tolerance and dialysis effectiveness in children. Adv Perit Dial 14:258–264 10649737

[20] Fischbach M, Terzic J, Becmeur F, Lahlou A, Desprez P, Battouche D, Geisert J (1996) Relationship between intraperitoneal hydrostatic pressure and dialysate volume in children on PD. Adv Perit Dial 12:330–334 8865930

[21] Rusthoven E, van der Vlugt ME, van Lingen- Bueren LJ, van Schaijk TC, Willems HL, Monnens LA, Schroder CH (2005) Evaluation of intraperitoneal pressure and the effect of different osmotic agents on intraperitoneal pressure in children. Perit Dial Int 25:352–356 16022091

[22] Sawaya AL, Damgov I, Menouer S, Terzic J, Peter Schmitt C, Zaloszyc A (2025) Intraperitoneal pressure measurements in children: a retrospective study. Nephrol Ther 21:13–22. 10.1684/ndt.2025.106 40066609

[23] Zaloszyc A, Schaefer B, Bartosova Medvid M, Edefonti A, Testa S, Paglialonga F, Shroff R, Doutey A, Walle JV, Lavaux T, Glady L, Delanghe J, Oyaert M, Friebus L, Damgov I, Fischbach M, Schmitt CP (2025) Randomized cross-over comparison of double mini-PET with standard versus adapted dwell volumes and dwell times in children on chronic peritoneal dialysis. Pediatr Nephrol. 10.1007/s00467-025-06993-x 41102531

[24] Dejardin A, Robert A, Goffin E (2007) Intraperitoneal pressure in PD patients: relationship to intraperitoneal volume, body size and PD-related complications. Nephrol Dial Transplant 22:1437–1444. 10.1093/ndt/gfl745 17308323

[25] Durand PY, Chanliau J, Gamberoni J, Hestin D, Kessler M (1992) Intraperitoneal pressure, peritoneal permeability and volume of ultrafiltration in CAPD. Adv Perit Dial 8:22–25 1361791

[26] Schmitt CP, Zaloszyc A, Schaefer B, Fischbach M (2011) Peritoneal dialysis tailored to pediatric needs. Int J Nephrol 2011:940267. 10.4061/2011/940267 PMC3132841 21761001

[27] Aranda RA, Romao Junior JE, Kakehashi E, Domingos W, Sabbaga E, Marcondes M, Abensur H (2000) Intraperitoneal pressure and hernias in children on peritoneal dialysis. Pediatr Nephrol 14:22–24 10.1007/s004670050005 10654324

[28] Outerelo MC, Gouveia R, Teixeira e Costa F, Ramos A (2014) Intraperitoneal pressure has a prognostic impact on peritoneal dialysis patients. Perit Dial Int 34:652–654. 10.3747/pdi.2012.00192 PMC4164410 25228214

[29] Giuliani A, Milan Manani S, Crepaldi C, Domenici A, Gastaldon F, Corradi V, Ronco C (2020) Intraperitoneal pressure in polycystic and non-polycystic kidney disease patients, treated by peritoneal dialysis. Blood Purif 49:670–676. 10.1159/000506177 32841944

[30] Sigogne M, Kanagaratnam L, Mora C, Pierre M, Petrache A, Marcus C, Fischbach M, Dramé M, Touré F (2020) Identification of the factors associated with intraperitoneal pressure in ADPKD patients treated with peritoneal dialysis. Kidney Int Rep 5:1007–1013. 10.1016/j.ekir.2020.04.012 PMC7335974 32647758

[31] Leung KC, Mahony S, Brown EA, Corbett RW (2024) Factors affecting intraperitoneal pressure (IPP) and its prognostic value in predicting leak risk and gastrointestinal symptoms in adult peritoneal dialysis patients: a systematic review and meta-analysis. J Nephrol 37:1767–1777. 10.1007/s40620-024-02091-7 39285125

[32] Li X, Ma T, Hao J, Song D, Wang H, Liu T, Zhang Y, Abi N, Xu X, Zhang M, Sun W, Li X, Dong J (2023) Novel equations for estimating intraperitoneal pressure among peritoneal dialysis patients. Clin Kidney J 16:1447–1456. 10.1093/ckj/sfad021 PMC10469109 37664572

[33] Fischbach M, Terzic J, Dangelser C, Schneider P, Roger ML, Geisert J (1998) Effect of posture on intraperitoneal pressure and peritoneal permeability in children. Pediatr Nephrol 12:311–314 10.1007/s004670050460 9655364

[34] Castellanos LB, Clemente EP, Cabanas CB, Parra DM, Contador MB, Morera JCO, Daly JA (2017) Clinical relevance of intraperitoneal pressure in peritoneal dialysis patients. Perit Dial Int 37:562–567. 10.3747/pdi.2016.00267 28698250

[35] Sobrino-Pérez A, Pérez-Escudero A, Fernández-Arroyo L, Dorado-García A, Martín-Alcón B, Gutiérrez-Martín C, Sánchez-Fonseca C, Barrios-Rebollo C, Pérez-Díaz V (2020) Intraperitoneal pressure: stability over time and validation of Durand’s measurement method. Perit Dial Int 41:427–431. 10.1177/0896860820973120 33250004

[36] Fischbach M, Terzic J, Chauve S, Laugel V, Muller A, Haraldsson B (2004) Effect of peritoneal dialysis fluid composition on peritoneal area available for exchange in children. Nephrol Dial Transplant 19:925–932. 10.1093/ndt/gfg518 15031351

[37] Yang SF, Liu CJ, Yang WC, Chang CF, Yang CY, Li SY, Lin CC (2015) The risk factors and the impact of hernia development on technique survival in peritoneal dialysis patients: a population-based cohort study. Perit Dial Int 35:351–359. 10.3747/pdi.2013.00139 PMC4443994 24584603

[38] Imholz AL, Koomen GC, Struijk DG, Arisz L, Krediet RT (1993) Effect of an increased intraperitoneal pressure on fluid and solute transport during CAPD. Kidney Int 44:1078–1085 10.1038/ki.1993.351 8264138

[39] Wang T, Heimburger O, Cheng H, Waniewski J, Bergstrom J, Lindholm B (1997) Effect of increased dialysate fill volume on peritoneal fluid and solute transport. Kidney Int 52:1068–1076 10.1038/ki.1997.430 9328947

[40] Rippe B (2006) Is intraperitoneal pressure important? Perit Dial Int 26:317–319; discussion 411 16722023

[41] Zhang J, Song L, Ma Z, Sun L, Wang X, Liu D, Huang F, Man Y (2024) Intra-abdominal pressure and residual renal function decline in peritoneal dialysis: a threshold-based investigation. Ren Fail 46:2312535. 10.1080/0886022X.2024.2312535 PMC10851793 38321869

[42] Kopitko C, Medve L, Gondos T, Soliman KMM, Fulop T (2022) Mediators of regional kidney perfusion during surgical pneumo-peritoneum creation and the risk of acute kidney injury-a review of basic physiology. J Clin Med 11:2728. 10.3390/jcm11102728 PMC9142947 35628855

[43] Kopitko C, Rosivall L, Medve L, Gondos T, Soliman KM, Szabo Z, Pettendi E, Fulop T (2023) Pneumoperitoneum and acute kidney injury-an integrative clinical concept review. ASAIO J 69:e54–e65. 10.1097/MAT.0000000000001866 36521162

[44] Chen YW, Liao CT, Wu MY, Huang NJ, Cherng YG, Wu MS, Hsu YH, Chen CH (2024) Pressure induces peritoneal fibrosis and inflammation through CD44 signaling. Ren Fail 46:2384586. 10.1080/0886022X.2024.2384586 PMC11293264 39082695

[45] Kirkpatrick AW, Roberts DJ, De Waele J, Jaeschke R, Malbrain ML, De Keulenaer B, Duchesne J, Bjorck M, Leppaniemi A, Ejike JC, Sugrue M, Cheatham M, Ivatury R, Ball CG, Reintam Blaser A, Regli A, Balogh ZJ, D’Amours S, Debergh D, Kaplan M, Kimball E, Olvera C, Pediatric Guidelines Sub-Committee for the World Society of the Abdominal Compartment Syndrome (2013) Intra-abdominal hypertension and the abdominal compartment syndrome: updated consensus definitions and clinical practice guidelines from the World Society of the Abdominal Compartment Syndrome. Intensive Care Med 39:1190–1206. 10.1007/s00134-013-2906-z PMC3680657 23673399

[46] WSACS (2021) Consensus guidelines. https://www.wsacs.org/education/436/wsacs-consensus-guidelines-summary/ accessed 16–8–2025

[47] Ferreira AC (2023) Intraperitoneal pressure in peritoneal dialysis patients: a need for treatment individualization. Clin Kidney J 16:1367–1368. 10.1093/ckj/sfad140 PMC10469088 37664561

[48] Brown EA, Blake PG, Boudville N, Davies S, de Arteaga J, Dong J, Finkelstein F, Foo M, Hurst H, Johnson DW, Johnson M, Liew A, Moraes T, Perl J, Shroff R, Teitelbaum I, Wang AY, Warady B (2020) International Society for Peritoneal Dialysis practice recommendations: prescribing high-quality goal-directed peritoneal dialysis. Perit Dial Int 40:244–253. 10.1177/0896860819895364 32063219

[49] Teitelbaum I, Glickman J, Neu A, Neumann J, Rivara MB, Shen J, Wallace E, Watnick S, Mehrotra R (2021) KDOQI US commentary on the 2020 ISPD practice recommendations for prescribing high-quality goal-directed peritoneal dialysis. Am J Kidney Dis 77:157–171. 10.1053/j.ajkd.2020.09.010 33341315

[50] Zhu F, Merlo LR, Tisdale L, Villarama M, Yi J, Haq Z, Wang X, Grobe N, Fischer K, Plahey K, Lasher RA, Chamney P, Schiller B, Kotanko P (2025) Relationship between intraperitoneal volume and intraperitoneal pressure during peritoneal dialysis-a pilot study in adult patients. Physiol Rep 13:e70179. 10.14814/phy2.70179 PMC11898012 40071771

