# Supplementary material for: Intraperitoneal pressure measurements in children on peritoneal dialysis: a review and European practice survey
Source: Pediatr Nephrol. 2026 Mar 26;41(11):3705–12. doi: 10.1007/s00467-026-07233-6 (PMC13612655; doi:10.1007/s00467-026-07233-6)
Supplement: Supplementary file 1 [file 467_2026_7233_MOESM2_ESM.docx]

**Supplemental data**

**Supplemental Table 1** Overview on studies of the effect of IPP on PD efficiency and PD patient outcome

| **Consequences of increased IPP** | **Studies** | **References** |
| --- | --- | --- |
| Increased morbidity and mortality | IPP >18 cm H2O may induce discomfort and pain in pediatric and adult patients  In an observational study in 61 adults, IPP > 14 cm had an increased risk of enteric peritonitis.  In 54 adult PD patients IPP > 13 predicted switch to hemodialysis or death. | [4, 8, 15, 19]  [24, 28] |
| Risk of hernias/leaks | In 14 school children on CAPD, a higher IPP was associated with hernias (10.9 ± 2.6 cm versus 8.1 ± 2.6 cm).  In 49 adult PD patients, those with IPP ≥ 20 cm had more hernias and leaks. | [27]  [34] |
| Compromised pulmonary function | Demonstrated in critically ill infants with AKI and adults on chronic PD | [6, 7] |
| RKF loss | In 62 adults, an increased IPP was an independent risk factor for RKF loss. An increase in IPP of 1 cm H_2_0 increased the risk of rapid RKF decline by 1.68-fold. Best RKF preservation with an IPP < 15.7 cm H_2_0 | [41] |
| Decrease of UF | Negative correlation of IPP with UF rate in adults | [25, 34, 38] |
| Change in small solutes clearance | Reduced clearance of small molecules in adults and children with high IPP | [19, 33, 38, 39] |

*AKI - acute kidney injury, CAPD - continuous ambulatory peritoneal dialysis, IPP - intraperitoneal pressure, PD - peritoneal dialysis, RKF - residual kidney function, UF = ultrafiltration*

Supplemental Table 2: PD fluids used for IPPM (32 centers, multiple answers allowed)

| PD fluid | N, (%) |
| --- | --- |
| Double chamber PD fluid, bicarbonate | 26 (81.1%) |
| Double chamber PD fluid, lactate | 2 (6.2%) |
| Double chamber PD fluid, mixed buffer | 6 (18.7%) |
| Icodextrin | 3 (9.4%) |
| Glucose concentration: low | 15 (46.8%) |
| Glucose concentration: medium | 7 (21.9%) |
| Glucose concentration: high | 2 (6.2%) |
| Other: patients own usual prescription/other | 2 (6.2%) |
| Single chamber acidic PD fluid | 0 |

*IPPM - intraperitoneal pressure measurement, PD - peritoneal dialysis*

**Survey**

Personal:

- City/country
- Number of actual PD patients
- Personal experience with PD in years
- Are you: Working in a specific pediatric dialysis center/ working in an adult unit?
- Are you: Pediatric nephrologist/Adult Nephrologist?

If you are an adult Nephrologist: are you treating patients below 18 years?

Center

- Do you measure IPP in peritoneal dialysis patients at your center?
- Yes
- No, why:
- lack of time/stuff
- lack of evidence
- lack of experience
- should/will do it
- others (please specify)

IPP measurements in centers

- If you measure IPP, do you have a written protocol on IPP measurements?
- Yes: in your center, in your country?
- No
- Do you adapt the volume prescription to the IPP measurement?
- Yes
- No

Adaptation

- Do you have age or body size related upper limits of acceptable IPP to consider?
- Yes
- No
- How do you adapt the volume?
- In case IPP is above the upper limit I reduce dialysate fill volume
- In case IPP is below the upper limit I increase fill volume
- I only modify dialysate fill volume based on two independent IPPM
- I do not change PD based on IPPM
- other

IPPM System:

- Do you use a specifically manufactured visual scale system?
- Yes
- No (in case no, please specify)
- Which type of PD fluid (multiple answers):
- single chamber acidic PD fluid
- double chamber PD fluid, lactate / bicarbonate / buffer mixture / all buffers
- glucose concentration: low medium high
- icodextrin
- Other fluid (no PD fluid, please specify)

Frequency of IPP measurements

- Do you measure IPP regularly in all patients?
- Yes: if yes at what interval (1; 3, 6, 12 monthly, others)
- No
- In case you do not measure regularly you measure on which occasion:
- Start of PD
- Before increasing IPV
- In case of poor fill volume tolerance by the patient/caregivers
- In case of PD related complications (hernia, leaks….)
- Low PD efficiency
- Other (please specify)

Dwell features

- Do you perform
- Single point IPP measurement
- Serial IPP measurements with increasing IP volumes
- Volumes are
- absolute volume (e.g., actual day/night-time fill volume), please specify
- per BSA (e.g. 600 /1000 /1400 ml/m2), please specify
- Dwell(s):
- specifically intended for IPP measurement only
- combined with other measurement (e.g. with PET), please specify
- IPP measurement dwell time:
- Right after infusion (within 10 min)
- After x mins (please specify)
- At the end of the dwell

What is the upper limit accepted IPP (cm water)?

- Infants below 2 years:
- Children 2 to 7 years:
- Older children:
